# Soul of the Jukskei River: The Extent of Bacterial Contamination in the Jukskei River in Gauteng Province, South Africa

**DOI:** 10.3390/ijerph18168537

**Published:** 2021-08-12

**Authors:** Kousar Banu Hoorzook, Anton Pieterse, Lee Heine, Tobias George Barnard, Nickey Janse van Rensburg

**Affiliations:** 1 Process Energy Environment Technology Station (PEETS), Faculty of Engineering and Built Environment, University of Johannesburg, P.O. Box 17011, Doornfontein, Johannesburg 2028, South Africa; nickeyjvr@uj.ac.za; 2 Water and Health Research Centre, Faculty of Health Sciences, University of Johannesburg, P.O. Box 17011, Doornfontein, Johannesburg 2028, South Africa; apieterse@uj.ac.za (A.P.); lheine@uj.ac.za (L.H.); tgbarnard@uj.ac.za (T.G.B.)

**Keywords:** chemistry, Jukskei river, microbiology, molecular biology, water quality

## Abstract

River water quality is an important health issue as the water is utilised for drinking, domestic and agricultural use in developing countries. This study aimed to investigate the effect water from a major city has on the water quality of the Jukskei River that daylights in Johannesburg, South Africa. The river water samples were analysed for physio-chemical properties, microbiology, antibiotic resistance of bacterial isolates, genetic markers, and potentially toxic metals. Data analysis revealed increased electrical conductivity, total dissolved solids, and turbidity since 2010. Total Coliform and *Escherichia coli* detected were above the South African water quality guidelines for domestic, recreational, and irrigation purposes. Additionally, sodium, zinc, nickel, lithium, and lead exceeded the guidelines in domestic, recreational, and irrigation water. Pathogenic strains of *E. coli* (aEPEC, EHEC, EIEC, and EAEC) were isolated from the water. Various other potentially pathogenic organisms that have been implicated as causes of gastro-intestinal, and a wide range of other diseases, were also detected and demonstrated multiple levels of resistance to antibiotics tested. The results show that the river water is a potential health threat to downstream users. These results will feed into the environmental management action plan for Water for the Future (NGO group).

## 1. Introduction

Safe drinking water remains inaccessible to several million people around the globe. The ever-increasing human population places severe pressure on the quality and quantity of sources of fresh drinking water and limits access to it [1]. The World Health Organization (WHO) estimates about 1100 million people, globally, drink unsafe water, and the more significant part of diarrhoeal disease (88%) is attributed to unsafe water, and inadequate sanitation and hygiene [2].

In South Africa, the Jukskei River is one of the main rivers that confluence into the western Crocodile River basin. It is one of the three largest rivers draining the northern suburbs of the Witwatersrand and arises from an underground spring in the Bezuidenhout Valley, east of Johannesburg (Gauteng Province). It passes through a range of urban settlements such as Alexandra Township, Buccleuch, and Midrand before its confluence with the Crocodile River and flows into the Hartbeespoort dam [3]. Due to both formal and informal settlements in relation to the river, it has been subject to many water quality-related issues in the past, in particular bacterial contamination and other types of pollution such as industrial and mining effluent [4,5,6]. Since 1986, the Department of Water and Sanitation (DWS) has been monitoring the water quality. Previously, this river was characterised as having low pH (3–4) and high concentrations of sulfate, chloride, fluoride, sodium, and nitrate [7]. The impact of the mining activities in the surrounding areas is possibly associated with high sulfate concentrations and low pH [7]. The presence of high-density informal settlements near Alexandra Township has been shown to increase nutrient concentrations, sodium, chloride, and potassium [3,5].

Since DWS only monitors fundamental microbiological analysis (Total Coliform and *Escherichia coli*), we hypothesised that bacteria isolated from the Jukskei River would be resistant to one or more antimicrobials and be multidrug-resistant based on the occurrence of increased antimicrobial resistance of potentially pathogenic bacteria isolated from various sources in South Africa [8,9,10]. Recent publications regarding antimicrobial resistance of potential pathogens isolated from environmental water sources in South Africa [8,9,10] focused on the antimicrobial resistance of targeted bacterial species like *Staphylococcus aureus*, *Campylobacter jejuni*, and vancomycin-resistant enterococci. In addition, the present study investigated the antimicrobial resistance of a wider variety of potential bacterial pathogens not previously studied.

The aim of the current study was to investigate the effect water from the city of Johannesburg (Gauteng, South Africa) has on the Jukskei River water quality after it daylights from the city centre. The specific objectives were to identify (a) what the chemical pollution levels were, (b) identify potentially pathogenic bacteria and determine their antibiotic resistance profile, and (c) if the river water could be used directly for agricultural and recreational purposes at Victoria yards (Lorenzville, Gauteng Province, Johannesburg, South Africa) where the river daylights.

## 2. Materials and Methods

### 2.1. Study Area and Sampling Site

**Figure 1:**
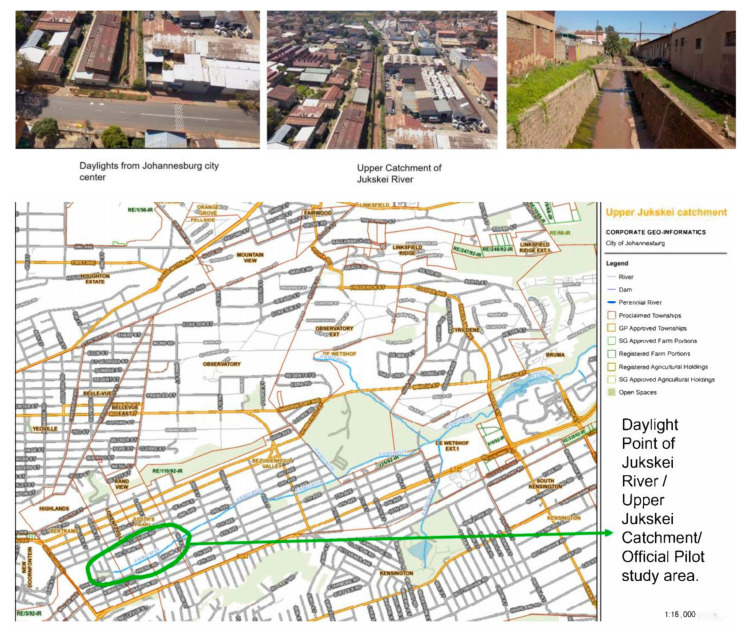
Drone visual and Geographical location of the upper Jukskei catchment study area (Courtesy of Joel Cruz and Water for the future).

The Jukskei River is one of the ten river catchments in Metropolitan Johannesburg and forms part of the catchment of the Limpopo River, which flows into the Indian Ocean. It flows north through the Bezuidenhout Valley, whereby the river is covered by stormwater culverts [5]. It then flows through several residential areas and informal settlements with no or limited access to municipal services. The Jukskei passes Alexandra, an informal township—a severely overpopulated area, which creates pressure on infrastructure and blocked sewers, causing overflow into the river [3]. The Jukskei flows in a northerly direction where it joins the Crocodile River, which then flows into the Hartbeespoort Dam. Three significant tributaries that join the Jukskei before it enters the Crocodile River are the Braamfontein Spruit, Klein Jukskei Spruit, and the Modderfontein Spruit [3]. The Jukskei River catchment receives effluent from industries and runoff in vast amounts from illegal, unmanaged waste dumps, and agricultural practices [11]. The Jukskei River catchment is largely urbanised and industrialised (Figure 1) [12].

Two surface water samples (upstream and downstream) were collected each month from June to August 2018 (winter and autumn season) at the section Jukskei River which runs through Victoria Yards (Lorenzville, Central Johannesburg, South Africa). These sites were selected because it was the only sites accessible to sample the daylight section of the upper Jukskei River catchment. In this section, Victoria yards want to recycle the river water for agricultural and recreational purposes. Figure 1 provides drone pictures of the sampling sites and the geographical location of the catchment area.

### 2.2. Sampling Collection

Six water samples were collected in sterile 250 mL sampling bottles with sodium thiosulphate and kept at 4 °C en route to the laboratory. The water samples were sent to SPECTRAU within the University of Johannesburg (UJ) to analyse the heavy metals, also referred to as potentially toxic elements using inductively coupled plasma mass spectrometry (ICP-MS). Water samples were sent to UJ Water and the Health Research Centre (WHRC) for microbiology analysis.

The following physio-chemical parameters were analysed in this study, water temperature, pH, electrical conductivity, total dissolved solids, and turbidity. The physio-chemical analysis for temperature (°C) (HI98129 pH/Conductivity/temperature, Hannah SA, Luxembourg), electrical conductivity (EC) (µs/m) (Hannah SA, Luxembourg), pH (Hannah SA, Luxembourg), and total chlorine (mg/L) (Cyberscan TB1000, Eutech, Thermo Fisher Scientific, Waltham, MA, USA) was performed on-site. Before taking measurements, the instruments were calibrated according to the manufacturer’s instructions. Turbidity (NTU) (YSI 900 Single Parameter Colorimeter, YSI, Yellow Springs, OH, USA) was tested in the laboratory. Total dissolved solids (TDS) were calculated using the calculation: $\mathrm{TDS}\;\left(\mathrm{mg}/L\right)=\left[\mathrm{EC}\;\left(µS/m\right)\times 0.001\;\left(\mathrm{dS}/m\right)\right]\;[\mathrm{EC}\left(\mathrm{dS}/m\right)\times 640\;(\mathrm{EC}<5\mathrm{dS}/m)]$ [13].

### 2.3. Microbial Analysis of Water

#### 2.3.1. Isolation of Total Coliforms and *E. coli*

Samples were analysed within three hours of collection for bacterial quality using the Colilert^®^ Quanti-Tray/2000^®^ (IDEXX, Westbrook, ME, USA). Enumeration of *E. coli* from the water was carried out using 100 mL of water according to the manufacturer’s instructions. The Colilert^®^ Quanti-Trays^®^/2000 were incubated for 18 h at 35 °C. After incubation, the Quanti-Trays^®^/2000 were examined under a long wave (366 nm) ultraviolet light, and wells that turned both yellow and fluoresced were counted as *E. coli* positive (IDDEX, Westbrook, ME, USA) [14].

#### 2.3.2. Isolation and Identification of Associated Bacteria

Water Samples were further cultured onto deoxycholate citrate agar (DCA), thiosulphate bile-salts sucrose agar (TCBS), and sorbitol MacConkey agar (SMAC) (Oxoid, Hampshire, UK) at 37 °C aerobically for 24–48 h.

The resulting isolates were cultured onto Müller Hinton Agar (Oxoid, Hampshire, UK) at 37 °C for 18–24 h. Samples were also cultured in duplicate on *Campylobacter* blood-free selective agar and incubated at 42 °C (one aerobically and in 5–10% carbon dioxide). Selected colonies from pure cultures were suspended in 0.45% saline solution (bioMérieux Inc., Marcy l’Etoile, France), and the density was determined using the VITEK^®^ 2 DensiCHEK™. Bacterial suspensions were adjusted to a 0.5–0.63 McFarland standard for Gram-positive cocci and Gram-negative bacilli, and a 1.8–2.2 McFarland standard for Gram-positive bacilli isolates.

Gram-positive bacilli and yeast isolated were identified using BCL and YST identification cards, respectively, and the VITEK^®^ 2 Compact automated system (bioMérieux Inc., Marcy l’Etoile, France). Antimicrobial susceptibility testing (AST) cards are unavailable for yeasts and Gram-positive bacilli. Therefore, these isolates were not subjected to AST in the present study. Gram-negative bacilli and Gram-positive cocci were identified using GN and GP identification cards and subjected to AST using N256 and P645 AST cards, respectively, and the VITEK^®^ 2 Compact system (bioMérieux Inc., Marcy l’Etoile, France). Details of categories and concentration ranges of antimicrobials tested using the AST-P645 and AST-N256 cards are shown in Tables S1 and S2 in the supplementary material.

The analysis was completed within 24 h, and isolates were identified using the VITEK^®^ 2 Compact System software. Minimum inhibitory concentrations (MICs) for each antimicrobial tested against each isolate were interpreted as either susceptible (S), intermediately resistant (I), or resistant (R). Isolates were then defined as exhibiting multidrug resistance (MDR), extensive drug resistance (XDR), or pan drug resistance (PDR) according to the guidelines set out by Magiorakos et al. [15].

### 2.4. Molecular Detection of Identified Bacteria

#### 2.4.1. DNA Extraction and Multiplex Polymerase Chain Reaction (m-PCR)

The *E. coli* results from the Colilert^®^ Quanti-Tray^®^/2000 were further classified as diarrhoeagenic or commensal *E. coli*. DNA was extracted as reported by Omar et al. [14], and 11 gene m-PCR was performed as written by Omar et al. [16]. Briefly, a total of 2 mL of the media was removed from up to ten positive *E. coli* wells of the Colilert Quanti-Trays^®^/2000 with sterile 1 mL Neomedic disposable syringes with a mounted needle (Kendon Medical Supplies, Sandton, South Africa) and aliquoted into 2 mL sterile Eppendorf tubes. The tubes were centrifuged for 2 min at 13,000× *g* to pellet the cells and the supernatant was discarded. DNA was extracted from the collected bacterial cells using an adapted version of the guanidium thiocyanate/silica method and homemade spin columns reported by Omar et al. [14]. DNA was eluted from the celite with 100 µL Qiagen^®^ elution buffer (Southern Cross Biotechnology^®^, Randburg, South Africa). The extracted DNA was used as a template in all PCR reactions.

All m-PCR reactions were performed in a Biorad Mycycler^TM^ (Dubai, United Arab Emirates) thermal cycler in a total reaction volume of 20 µL. A hotstart multiplex PCR kit (Qiagen^®^, Hilden, Germany) was used for the m-PCR protocol. Each reaction consisted of 1X Qiagen^®^ PCR multiplex mix (containing HotstartTaq^®^ DNA polymerase, multiplex PCR buffer, and dNTP mix); 2 µL of the primer mixture (0.1 lM of *mdh* and *lt* primers [Forward (F) and reverse (R)), 0.2 lM of *ial*, *eagg* primers, *astA* primers, *bfp* primers, and *gapdh* primers (F and R), 0.3 lM of *eaeA* and *stx2* primers (F and R), 0.5 lM of *stx1* and *st* primers (F and R) [16]; 2 µL of sample DNA, 1 µL of *gapdh* cDNA and 5 µL PCR grade water. The reactions were subjected to an initial activation step at 95 °C for 15 min, followed by 35 cycles consisting of denaturing at 94 °C for 45 s, annealing at 55 °C for 45 s, extension at 68 °C for 2 min and final elongation at 72 °C for 5 min. DNA was visualised using a 2.5% (*w*/*v*) agarose gel in TAE buffer (40 mmol/L Tris-acetate; 2 mmol/L EDTA, pH 8.3) with 0.5 lg mL^−1^ ethidium bromide. Electrophoresis was performed for 1–2 h in an electric field strength of 8 V cm^−1^ gel and the DNA was visualised with UV light (Syngene, Cambridge, UK). This procedure was followed for all the experiments except where stated differently. The relative sizes of the DNA fragments were estimated by comparing their electrophoretic mobility with that of the standards run with the samples on each gel, either with a 1 kB or 100 bp marker (Fermentas, Waltham, MA, USA) [16].

The *Vibrio* spp. was further classified as *Vibrio cholerae* 01 or 0139 as reported by Ntema et al. [17]. The m-PCR targeted *V. cholerae* O1 and *V. cholerae* O139 *rfb* genes, *ctxA* (cholera toxin), and the 16S rRNA gene. The *Vibrio* spp. PCR product Genomic DNA was sent to Inqaba Biotechnical Industries S.A. (Pretoria, South Africa) a commercial NGS service provider, for sequencing. Briefly, genomic DNA samples were PCR amplified using a universal primer pair 341F and 785R—targeting the V3 and V4 region of the 16S rRNA gene. The resulting amplicons were gel purified, end-repaired and Illumina-specific adapter sequences were ligated to each amplicon. Following quantification, the samples were individually indexed, and another purification step was performed. Amplicons were then sequenced on the Illumina MiSeq platform, using a MiSeq v3 (600 cycle) kit. 20 Mb of data (2 × 300 bp long paired-end reads) were produced for each sample. The BLAST-based data analysis was performed using an Inqaba in-house developed data analysis pipeline.

#### 2.4.2. Potentially Toxic Elements

A total of six water samples was analysed for potentially toxic elements using (ICP-MS). The potentially toxic elements analysed were lithium, beryllium, boron, sodium, aluminium, vanadium, chromium, manganese, iron, copper, nickel, cobalt, zinc, arsenic, selenium, molybdenum, cadmium, uranium, and lead.

### 2.5. Statistical Analysis

Descriptive statistical analysis of the physio-chemical and microbiological parameters was analysed to summarise the data obtained for the three months. Variations in physical chemistry and microbiological parameters across sampling for the 3 months were analysed by one-way analysis of variance (ANOVA). The statistical analysis was performed using GraphPad Prism 9 (GraphPad Software, San Diego, CA, USA).

## 3. Results and Discussion

### 3.1. Physio-Chemical Analysis

All the analysed physio-chemical parameter levels for recreational and irrigation water were within acceptable limits according to the South African Water Quality guidelines [18]. No significant statistical variation was observed (*p* < 0.05) for the physio-chemical parameters between the two sites (upstream and downstream). The water temperature ranged from 13.07 to 19 °C (mean 15.9 °C) and did not show any significant variation (*p* = 0.184) for June–August 2018. Although the water temperature is highly variable, it is an important parameter in aquatic systems as it affects the rate of metabolic activities in organisms, it can also increase the toxicity of certain chemicals in the water [18,19]. The temperature recorded was within the range for aquatic ecosystems in the Jukskei catchment of 11.9 to 29.9 °C [5]. The pH ranged from 7.11 to 7.38 (mean 7.31) and did not show any significant variation (*p* = 0.576). The pH stayed relatively consistent at the Jukskei River from 2009 as reported by Matowanyika et al. [5], with the same trend in pH also reported in the Senqu–Orange River and Mohokare River [20]. The electrical conductivity ranged from 420 to 472 µS/m (mean 441 µS/m) with no significant variation (*p* = 0.148) shown. For most freshwaters, EC ranges from 10 to 1000 µS/m, and elevated levels of above 1000 µS/m can be seen in polluted water that receives large volumes of land runoff [21]. In streams and rivers, conductivity is affected by various factors such as type of soils, bedrocks, presence of inorganic dissolved solids sewage, and wastewater. The EC increased from 1994 to 2008 (30–80 µS/m) as reported by Matowanyika et al. [5] to 441 µS/m in the current study. Total dissolved solids (TDS) ranged between 269 to 302 mg/L (mean 282 mg/L) and did not show significant variation (*p* = 0.193). TDS increased from 10–104 mg/L in 2010 to 260–302 mg/L [5]. TDS is directly proportional to the concentration of EC and this relationship concurs with the report from Chatanga et al. [20]. Total chlorine was 0 g/L and turbidity ranged from 35.9 to 85.7 NTU (mean 50.4 NTU) (Table 1). Turbidity did not show significant variation (*p* = 0.153) for June to August 2018. However, the turbidity increased from 27.1 NTU in 2010 to a mean of 50.3 NTU [5]. Turbidity normally increases in South African rivers during the rainy season. However, June–August is cold and dry. Increased microbial load and anthropogenic activities such as road and bridge construction can result in increased levels of turbidity and TDS [5,22].

### 3.2. Microbiology Analysis

#### 3.2.1. Isolation of TC (Total Coliforms) and *E. coli*

Six water samples were analysed with the Colilert^®^ Quanti-Tray^®^/2000 method for the presence and microbial load of TC and *E. coli*. The TC counts ranged from 3.2 × 10^6^ to 4.0 × 10^7^ Most Probable Number (MPN/100 mL) (mean 1.8 × 10^7^ MPN/100 mL) and did not show any significant statistical variation (*p* = 0.103) for June–August 2018. *E. coli* counts ranged between 8.9 × 10^5^–4.0 × 10^6^ MPN/100 mL (mean 2.1 × 10^6^ MPN/100 mL) (Table 1) and showed significant statistical variation (*p* = 0.048). These values are above the South African Water Quality guidelines [19] for irrigation and recreational use. No significant statistical variation was observed (*p* < 0.05) for TC and *E. coli* between the two sites (upstream and downstream). The *E. coli* values detected in 2003 were in the range of 3 × 10^5^ cfu/mL and in 2010 the values were 1 × 10^5^ cfu/mL [5]. Microorganisms have been reported to be positively related to turbidity, total suspended solids, and TDS [20]. This can be seen with the high levels of turbidity and TDS reported in Section 3.1. An important factor that should be considered in disinfection is the turbidity of the water; the reason is that when water contains colloidal particles, they may shield the microorganisms from the action of the disinfection or react with the chlorine and in this way prevent effective disinfection [23]. *E. coli* is generally regarded as a specific microbial indicator of faecal pollution from humans and warm-blooded animals [24] but also have various highly pathogenic types within the group capable of causing diseases such as diarrhoea, dysentery, kidney failure, bladder infections, septicaemia, pneumonia, and meningitis [25,26].

#### 3.2.2. Isolation and Identification of Associated Bacteria

##### Microbiology Analysis

The WHO released its priority list of antibiotic-resistant bacteria grouped into three tiers: critical high priority (*Acinetobacter baumannii*, *Pseudomonas aeruginosa*, *Klebsiella pneumoniae*, *Escherichia coli*, *Enterobacter* spp., *Serratia* spp., *Proteus* spp., *Providencia* spp., and *Morganella* spp.), high priority (*Enterococcus faecium*, *Staphylococcus aureus*, *Helicobacter pylori*, *Campylobacter*, *Salmonella* spp., and *Neisseria gonorrhoeae*) and medium priority (*Streptococcus pneumoniae*, *Haemophilus influenzae*, and *Shigella* spp.) [27] Within these tiers, a group of bacterial pathogens commonly referred to as the ‘’ESKAPE’’ pathogens (*Enterococcus faecium*, *Staphylococcus aureus*, *Klebsiella pneumoniae*, *Acinetobacter baumannii*, *Pseudomonas aeruginosa,* and *Enterobacter* spp.) contribute significantly to the burden of disease in both developed and developing countries due to their ability to carry and easily acquire multiple antibiotic-resistant genes [28,29,30,31]. The organisms are opportunistic pathogens implicated in both nosocomial infections and community-acquired outbreaks [32]. These pathogens are of concern especially for vulnerable groups using untreated water. In Table 2, 129 isolates were identified and confirmed with the VITEK^®^ 2 Compact System, these isolates belong to 35 different bacterial species. From Table 2, it can be seen that four out of six of these ‘’ESKAPE’’ pathogens were present in the water, namely *Acinetobacter baumannii*, *Pseudomonas aeruginosa*, *Klebsiella pneumoniae*, and *Staphylococcus aureus*. The table further shows potential infections various other organisms can cause such as meningitis, gastroenteritis, urinary tract infections, corneal infections, bloodstream infections, soft tissue, and skin infections, etc.

**Figure 2:**
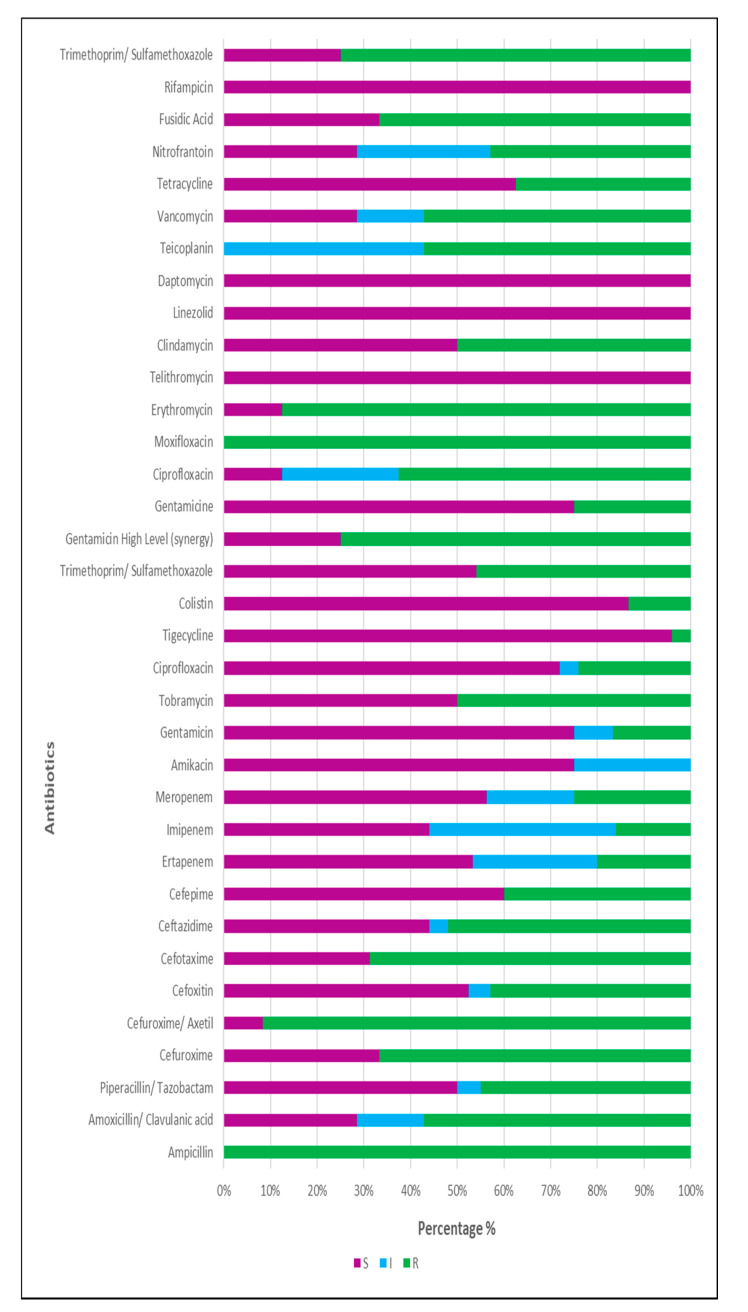
Percentage of bacterial isolates (*n* = 33) that are susceptible (S), intermediately resistant (I), or resistant (R) to each of the listed antibiotics.

Figure 2 provides the antibiotic resistance profile for the 33 isolates (16 bacterial species) isolated and identified from the water samples. The bacteria selected are from a community of bacteria that are present in the water; the antibiotic profile does not indicate that all the genus or species in the water are resistant or susceptible to the antibiotics, only the selected colonies have the below antibiotic profile (Tables S3 and S4).

High levels of resistance were noted in the 25 isolates subjected to antimicrobial susceptibility testing (Figure 2). Of these, 8% (2/25) showed resistance to none of the antimicrobials in any of the categories tested, and 20% (5/25) showed resistance to antimicrobials in either one or two categories. A further 72% (18/25) were identified as MDR isolates and were resistant to one or more antimicrobials in between three and ten categories of antibiotic drugs. No XDR or PDR isolates were identified after susceptibility testing and interpretation. Of the 18 MDR isolates, 44% (8/18) were categorised as extended-spectrum β-lactamase (ESBL) producers, 33% (6/18) of isolates showed a high level of Amp C resistance, and 22% (4/18) were shown to be extended-spectrum carbapenemase producers or carbapenem impermeability. The resistance of two isolates to colistin is worrying as this is an antimicrobial of last resort for the treatment of MDR and XDR organisms [123,124]. The results of the present study correlate with those of Müller et al. [125], which show these highly antimicrobial-resistant organisms to be present in wastewater and environmental water sources. The majority of MDR isolates were clinically significant species—i.e., those capable of causing infection in humans. This is problematic as treatment of infection with organisms that are resistant to antibiotics is more complex and increases morbidity and mortality of infected patients [126]. The most common antibiotic-resistant, clinically relevant gram-negative species comprise ESBL- and carbapenemase-producing Enterobacteriaceae, such as *Klebsiella pneumoniae* and *E. coli,* as well as non-fermenters such as *Pseudomonas aeruginosa* and *Acinetobacter baumannii* [127], some of which form part of the ‘’ESKAPE’’ pathogens and the WHOs priority list of antibiotic-resistant bacteria. The high prevalence of multidrug resistance (MDR) in the isolates subjected to AST is of concern as the bacteria isolated are responsible for community and nosocomial infection. Antimicrobial resistance increases patient morbidity and mortality and increases [126] the financial burden of disease, which is especially significant in low-income countries such as South Africa [128]. MDR was defined as non-susceptibility to one or more antimicrobials in three or more drug categories. Extensive drug resistance (XDR) was defined as non-susceptibility to one or more antimicrobials in all but two drug categories. Pan drug resistance (PDR) was defined as non-susceptibility to one or more antimicrobials in all drug categories. These definitions do not include intrinsic resistances displayed by some genera to specific antibiotics, and, therefore, only consider acquired resistance [15].

##### Molecular Biology Analysis

All six water samples detected the following potential pathogenic *E. coli*: Atypical Enteropathogenic *E. coli* (EPEC), Enterohaemorrhagic *E. coli* (EHEC), Enteroinvasive *E. coli* (EIEC), Enteroaggregative *E. coli* (EAEC), and toxin astA that can be detected in both commensal and pathogenic *E. coli*.

*E. coli* is one of the best known and earliest described human opportunistic pathogenic bacteria. It is also a specific microbial indicator of faecal pollution from humans and warm-blooded animals [24]. *E. coli* is divided into intestinal pathogens [Diarrhoegenic *E. coli* (DEC)] and extraintestinal *E. coli* (ExPEC), causing a variety of infections in both animals and humans, including urinary tract infections, meningitis, and septicaemia [129,130]. There are at least eight DEC pathotypes and four ExPEC [131,132]. Eight pathotypes are diarrhoeagenic, based on their pathogenic mechanisms; however, five DEC were selected for this study based on their importance for surface-water pathogenicity. These include the EPEC, ETEC, EHEC, EAEC, and EIEC [133,134].

The *Vibrio cholerae* was confirmed with m-PCR and 16S rDNA sequencing as a non-pathogenic environmental isolate. *Vibrio* spp. has been associated with domestic sewage in the river and most *Vibrio* spp. enumerated are of animal origin [135].

##### Potentially Toxic Elements

A total of six water samples was sent to SPECTRAU within the UJ (the University of Johannesburg) to analyse the potentially toxic elements. Table 3 provides the results compared to the specified drinking, recreational, and irrigation water guidelines [18,19]. The results show that the potential toxigenic elements were within the DWS water quality guidelines for irrigation, recreation, and drinking purposes, except for Li, Ni, Zn, Pb, and Na for irrigation, recreation, and drinking purposes [18,19].

The concentration of uranium is very low, which means that the water is non-radioactive. Sodium is above the guideline for drinking water and irrigation water. The guideline for drinking water is <200 µg/L, and the guideline for irrigation water is 2000 µg/L, but the average concentration for sodium is 45,545 µg/L. Sodium is not suitable for irrigation; it causes a shortage of calcium and potassium in the soil. These are essential nutrients for plants. The deficiency of these nutrients results in the poor growth of plants. Sodium is also bad for the body if it is used excessively. It causes nausea, vomiting, and can cause heart failure [2]. According to da Silva et al. [3], high levels of sodium, above 400 mg/L with an alkaline pH and an increased level of phosphate, is a suitable environment for Faecal Coliforms, thus elevating the number’s present in the Jukskei River. The contribution of increased levels of sodium in the water could be from soaps and sodium salts [3].

Zinc is above the guideline for drinking, recreation, and irrigation water. The guideline for drinking water is <5 µg/L, and the guideline for irrigation water is 100–2000 µg/L, whereas the average concentration for zinc is 116.68 µg/L. Toxicity in humans may occur if zinc concentration approaches 400 mg/kg and 3 mg/L in soil and water, respectively. This is characterised by symptoms of irritability, muscular stiffness and pain, loss of appetite, and nausea [22,136].

Lead is above the guideline for drinking water. The guideline for drinking water is <10 µg/L, whereas the concentration of lead in different samples was higher than the guideline. It has concentrations of 15.2 µg/L and 21.6 µg/L. Lead is highly toxic, and its widespread use has caused extensive environmental contamination and health problems in many parts of the world [22]. The common symptom of lead poisoning is anaemia because lead interferes with the formation of haemoglobin, and prevents iron uptake. Higher levels of lead may induce permanent brain damage and kidney dysfunction. Over time, the lead substitutes Ca in the bones which acts to store the lead. Then in old age, the lead is reactivated by the slow dissolution of the bones [22].

Lithium is above the guideline for irrigation, recreation, and drinking. The guideline for irrigation and recreation is <2500 µg/L, whereas the average concentration is 2710 µg/L. Lithium is a naturally occurring element in drinking water mainly originating from weathering of minerals in the subsurface. Lithium, in most of the world’s major rivers, is derived predominantly from silicate weathering, and the fraction is derived from carbonate rocks [137].

Nickel is above the guideline for drinking, irrigation, and recreation water. The guideline is 200–2000 µg/L, whereas the average concentration for nickel was 2279 µg/L. Nickel is noted in exceptional cases of release from natural or industrial nickel deposits in the ground. Nickel has an extensive range of carcinogenic mechanisms, which include regulation of transcription factors, controlled expression of specific genes, and generation of free radicals [22]. Nickel is implicated in regulating the expression of specific long non-coding ribonucleic acids (RNA). It has also been demonstrated that nickel can generate free radicals, contributing to carcinogenic processes [138].

Between 1987 and 1990, the Urban Renewal Plan was implemented at the Jukskei River to reduce pollution load. A water reticulation system, water-borne sewage pipes, electrical reticulation, and ablution facilities were provided to all dwelling units in Alexandra Township within the Jukskei River catchment. This increased the number of residents, thus increasing the urban runoff and pollution load in the river [139]. Furthermore, a potential source for water pollution includes wastewater effluent and hazardous waste from manufacturing industries, and the collapse of the stormwater and wastewater infrastructure, which lies above each other, thus flowing as one when flowing out of the city centre [140]. This has several environmental impacts, such as excess storm-runoff which reaches the river bringing along the pollutants and toxicants from neighbouring land uses including industry or mining [3].

## 4. Conclusions

The aim was to investigate the effect water from the city of Johannesburg has on the water quality flowing to the lower Jukskei River catchment. The results show that even though the physical chemistry was within the South African guideline limit, the detection levels have increased from 2010 to 2018 for EC, TDS, and turbidity. Microorganisms have been reported to be positively related to turbidity and TDS. This can be seen with the TC and *E. coli* and the identification of associated bacteria. The TC and *E. coli* values were above the DWS 1996 guidelines for recreational and agricultural use and SANS 241-2:2015 guidelines for drinking water. Potential pathogenic *E. coli*, the ‘’ESKAPE’’ pathogens, and various associated pathogenic organisms have also been detected that can cause gastrointestinal diseases, traveller’s diarrhoea, skin, wound, and urinary tract infections, pneumonia, and various other diseases. These organisms detected have various levels of resistance when subjected to antimicrobial susceptibility testing. Most published articles on river water quality analysis analyse the microbial indicators to determine the extent of the pollution load and assume that pathogenic bacteria are present. In this study, results show the importance of microbial pollution in the Jukskei River in which the potential source includes domestic sewage and wastewater effluent.

For the potentially toxic elements analyses, Lithium, Nickel, Zinc, Lead, and Sodium were above the South African irrigation, recreation, and drinking water quality guidelines if the water is used for these purposes. Lead is highly carcinogenic, and Nickel causes cell damage and affects carcinogenic processes. The toxicity of potentially toxic elements could be acute, while others could be chronic after long-term exposure.

The water quality of river systems is crucial, given that rivers are systems that connect communities in space. Activities upstream have consequences on downstream users and systems. The Jukskei River water is a potential threat to people using the river as a source of recreational, agricultural, and domestic water. Furthermore, bioaccumulation of pollutants can occur in the food chain. The water is also a threat to aquatic ecosystem health and integrity.

## Figures and Tables

**Table 1:** Descriptive statistics for the physio-chemical and microbiology results from June to August 2018.

Test	Unit	Min	Max	Mean	Standard Deviation
Physio-Chemical Analysis					
pH		7.11	7.38	7.31	0.09
Turbidity	NTU	35.9	85.7	52.4	5.5
Electrical conductivity	µS/m	420	472	441	15.8
Temperature	°C	13.8	19	15.9	0.64
Total dissolved solids	mg/L	269	302.1	282	9.91
Microbiology Analysis					
Total coliforms	MPN/100 mL	4.1 × 10^6^	3.9 × 10^7^	1.8 × 10^7^	3.5 × 10^6^
*Escherichia coli*	MPN/100 mL	8.9 × 10^5^	4.0 × 10^6^	2.1 × 10^6^	1.3 × 10^5^

**Table 2:** List of bacterial species (*n* = 35) identified and confirmed with the VITEK^®^ 2 Compact System.

Organism	Nr. of Isolate/s per Specie	Associated Human Disease	Reference
*Acinetobacter baumannii*	2	Bloodstream infection, Endocarditis, Meningitis, Ophthalmitis/keratitis, Peritonitis, neumonia, Soft tissue/skin infections, Urinary tract infections, Wound infections	[33,34,35]
*Acinetobacter lwoffii*	1	Gastroenteritis, Meningitis, Pneumonia, Septicaemia, Skin infections, Urinary tract infections	[36]
*Aerococcus* *viridans*	2	Bacteraemia, Cellulitis, Endocarditis, Soft tissue infection, Urinary tract infections	[37,38,39,40,41]
*Aeromonas* *sobria*	10	Bacteraemia, Sepsis, Traveller’s diarrhoea, Urinary tract infections	[42,43]
*Aeromonas* *hydrophila*	22	Gastroenteritis, Peritonitis, Sepsis, Septicaemia, Traveller’s diarrhoea	[42,43]
*Aeromonas* *caviae*	22	Keratitis, Traveller’s diarrhoea, Urinary tract infections	[42,43]
*Bacillus* *pumilus*	1	Catheter infection, Necrotic skin infection, Sepsis, Septic arthritis	[43,44,45,46,47]
*Candida* *lusitaniae*	1	Fungemia and Prosthetic joint infection	[48,49]
*Citrobacter braakii*	4	Bacteraemia and Urinary tract infections	[50,51]
*Citrobacter freundii*	4	Bacteraemia, Diarrheal, Urinary tract infections	[52,53,54]
*Comamonas* *testosterone*	1	Appendicitis, Bacteraemia, Catheter-related infection, Cholesteatoma, Endocarditis, Gastroenteritis	[55,56,57,58]
*Enterobacter asburiae*	1	Bloodstream infections, Osteomyelitis, Pneumonia, Soft-tissue and skin infections, Urinary tract infections	[59]
*Enterococcus faecalis*	8	Sepsis and Urinary tract infection	[60]
*Enterococcus* *hirae*	1	Bacteraemia, Pyelonephritis, Urinary tract infection	[61,62,63,64]
*Escherichia coli*	14	Diarrheal, Gastroenteritis, Urinary tract infections	[53,54,60,65]
*Gemella* *haemolysans*	1	Bacteraemia, Brain abscess, Endocarditis	[66,67,68]
*Gemella* *morbillorum*	1	Brain abscess, Endocarditis, Keratitis	[69,70,71]
*Klebsiella* *pneumoniae*	1	Bacteraemia, Cystitis, Meningitis, Pneumonia, Pyelonephritis, Pyogenic liver abscess, Sepsis, Septicaemia, Soft-tissue infections, Urinary tract infections, Wound infections	[72,73,74,75]
*Kluyvera* *ascorbate*	2	Diarrheal, Sepsis, Urinary tract infection	[76,77]
*Kluyvera* *cryocrescens*	1	Bacteraemia, Cholecystitis, Sepsis, Soft-tissue infections	[76,77,78]
*Micrococcus* *luteus*	4	Brain abscess, Meningitis, Myocarditis, Periprosthetic joint infection, Pyogenic liver abscess	[79,80,81]
*Providencia* *alcalifaciens*	1	Diarrheal, Gastroenteritis, Keratitis	[82,83,84,85]
*Pseudomonas* *aeruginosa*	2	Respiratory tract infections, Soft-tissue infections	[86]
*Pseudomonas fluorescens*	2	Bloodstream infection and Meningitis	[87,88,89]
*Pseudomonas stutzeri*	2	Corneal infection, Endocarditis, Prosthetic joint infection, Urinary tract infection	[90,91,92,93,94]
*Raoultella planticola*	2	Bacteraemia, Bloodstream infections, Cholecystitis, Necrotizing fasciitis, Pancreatitis, Pneumonia, Urinary tract infection	[95,96,97,98,99]
*Serratia* *plymuthica*	2	Osteomyelitis, Sepsis, Urinary tract infection	[100,101]
*Shewanella* *putrefaciens*	2	Bacteraemia, Ear infection, Skin, and soft-tissue infections	[102]
*Staphylococcus aureus*	1	Endocarditis, Food poisoning, Meningitis, Osteomyelitis, Pneumonia, Prosthetic joint infection, Septic arthritis, Septic shock, Septic Thrombophlebitis, Skin and soft-tissue infections, Skin disease, Staphylococcal scalded skin syndrome, Systemic infections, Toxic shock syndrome, Urinary tract infections	[47,65,103,104,105,106,107]
*Staphylococcus* *auricularis*	1	Bacteraemia, Periprosthetic joint infection, Vaginitis	[108,109,110]
*Staphylococcus cohnii*	4	Bacteraemia, Meningitis, Urinary tract infections	[111,112,113]
*Staphylococcus* *haemolyticus*	2	Bloodstream infections, Endocarditis, Meningitis, Peritonitis, Prosthetic joint infection, Urinary tract infections, Vaginitis	[103,106,107,110,114,115]
*Staphylococcus* *vitulinus*	2	Bloodstream infections, Endocarditis, Pelvic inflammatory disease, Peritonitis, Prosthetic joint infection, Septic shock, Urinary tract infections, Wound infections	[114,116]
*Staphylococcus* *warneri*	1	Bloodstream infections, Discitis, Endocarditis, Infection of CSF shunts, Meningitis, Osteomyelitis, Peritonitis, Prosthetic joint infection, Sepsis, Subdural empyema, Urinary tract infections	[103,106,108,114,117,118,119,120,121]
*Vibrio cholerae*	1	Cholera and Gastroenteritis	[65,105,122]

**Table 3:** Results of the ICP-MS potentially toxic elements.

Sample Id	Li 7 µg/L	Be 9 µg/L	B 11 µg/L	Na 23 µg/L	Al 27 µg/L	V 51 µg/L	Cr 52 µg/L	Mn 55 µg/L	Fe 57 µg/L	Co 59 µg/L	Ni 60 µg/L	Cu 63 µg/L	Zn 66 µg/L	As 75 µg/L	Se 82 µg/L	Mo 98 µg/L	Cd 111 µg/L	Pb 208 µg/L	U 238 µg/L
Upstream 1st	4.097	<0.1	35.5	48,282	68.9	1.3	0.6	52.4	211	0.6	3.403	15.2	23.8	0.8	<0.1	0.5	<0.1	1.1	0.5
Downstream 1st	3.254	<0.1	45.0	49,581	77.1	1.4	0.6	50.6	173	0.6	3.121	25.0	37.6	0.6	<0.1	0.5	<0.1	0.9	0.7
Upstream 2nd	4.340	<0.1	12.7	39,586	85.3	1.5	0.6	51.8	220	0.6	3.470	28.5	267	0.7	<0.1	1.4	0.2	21.6	1.1
Downstream 2nd	4.507	<0.1	8.7	44,734	112	2.3	0.8	55.6	257	0.7	3.665	24.2	255	1.0	<0.1	1.4	0.2	15.2	1.0
Upstream 3rd	2.9	95.6	<0.1	54,168	74.3	1.2	0.8	53.1	166	0.6	5.0	10.2	40.3	0.3	<0.1	0.5	<0.1	1.2	0.7
Downstream 3rd	3.2	106.6	<0.1	46,958	64.8	1.1	0.7	54.3	221	0.5	7.8	9.6	48.7	0.2	9.4	0.5	<0.1	1.6	0.6
SANS 241 Drinking water Guideline (SABS, 2015)	<2400	*	*	<200	<300	*	<50	<100	<2000	*	<70	<2000	<5	<10	<40	*	<3	<10	<30
SA Irrigation water guideline (DWAF, 1996)	<2500	100–5000	500–1000	2000	5–20,000	100–1000	100	20–10,000	500–20,000	50–500	200–2000	200–5000	100–2000	100	20–50	10–50	10–50	200–2000	10–100

Note: * no value provided from guideline.

## Data Availability

Available at University of Johannesburg data repository, link https://figshare.com/s/330479b055d982d4065d.

## Supplementary Materials

The following are available online at https://www.mdpi.com/article/10.3390/ijerph18168537/s1, Table S1: Details of categories and concentration ranges of antimicrobials tested using the AST-P645 card, Table S2: Details of categories and concentration ranges of antimicrobials tested using the AST-N256 card, Table S3: Antimicrobial susceptibility profiles Gram-positive cocci subjected to AST, and Table S4: Antimicrobial susceptibility profiles Gram-negative bacilli subjected to AST.
