# Supplementary material for: Soul of the Jukskei River: The Extent of Bacterial Contamination in the Jukskei River in Gauteng Province, South Africa
Source: Int J Environ Res Public Health. 2021 Aug 12;18(16):8537. doi: 10.3390/ijerph18168537 (PMC8392637; doi:10.3390/ijerph18168537)
Supplement: Supplementary file 1 [file ijerph-18-08537-s001.zip › ijerph-1283948-supplementary.pdf]

Table S1. Details of categories and concentration ranges of antimicrobials tested using the AST-P645 card.

Category	Antimicrobial	Range
Cephalomycins	Cefoxitin Screen	Negative or Positive
Penicillins	Oxacillin	0.25 – 4 µg/mL
Non-Extended Spectrum Cephalosporins	Cefuroxime	1 – 64 µg/mL
	Cefuroxime Axetil	1 – 64 µg/mL
Aminoglycosides	Gentamicin High Level (synergy)	Susceptible or Resistant
	Gentamicin	0.5 – 16 µg/mL
Fluoroquinolones	Ciprofloxacin	0.5 – 8 µg/mL
	Moxifloxacin	0.25 – 8 µg mL
Lincosamides	Inducible Clindamycin Resistance	Negative or Positive
	Clindamycin	0.125 – 4 µg/mL
Macrolides	Erythromycin	0.25 – 8 µg/mL
	Telithromycin	0.25 – 4 µg/mL
Oxazolidinones	Linezolid	0.5 – 8 µg/mL
Lipopeptides	Daptomycin	0.12 – 8 µg/mL
Glycopeptides	Teicoplanin	0.5 – 32 µg/mL
	Vancomycin	0.5 – 32 µg/mL
Tetracyclines	Tetracycline	1 – 6 µg/mL
Nitrofuran	Nitrofurantoin	16 – 512 µg/mL
Fucidanes	Fusidic Acid	0.5 – 32 µg/mL
Carboxylic Acids	Mupirocin	1 – 512 µg/ mL
Ansamycins	Rifampicin	0.5 – 32 µg/ mL
Folate Pathway Inhibitors	Trimethoprim/ Sulfamethoxazole	10 – 320 µg/ mL

Table S2. Details of categories and concentration ranges of antimicrobials tested using the AST-N256 card.

**Category**	**Antimicrobial**	**Range**
Penicillins	Ampicillin	2 – 32 µg/mL
Penicillin and -Lactam Inhibitors	Amoxicillin/ Clavulanic acid	2/ 1 – 32/ 16 µg/mL
Anti-Pseudomonal Penicillins	Piperacillin/ Tazobactam	4/ 4 – 128/ 4 µg/mL
Non-Extended Spectrum Cephalosporins	Cefuroxime	1 – 64 µg/mL
	Cefuroxime-Axetil	1 – 64 µg/mL
Cephalomycins	Cefoxitin	4 – 64 µg/mL
Extended Spectrum Cephalosporins	Cefotaxime	1 – 64 µg/mL
	Ceftazidime	1 – 64 µg/mL
	Cefepime	1 – 64 µg/mL
Carbapenems	Ertapenem	0.5 – 8 µg/mL
	Imipenem	0.25 – 16 µg/mL
	Meropenem	0.25 – 16 µg/mL
Aminoglycosides	Amikacin	2 – 64 µg/mL
	Gentamicin	1 – 16 µg/mL
	Tobramycin	1 – 16 µg/mL
Fluoroquinolones	Ciprofloxacin	0.25 – 4 µg/mL
Glycylcyclines	Tigecycline	0.5 – 8 µg/mL
Polymixins	Colistin	0.5 – 16 µg/mL
Folate pathway inhibitors	Trimethoprim/ Sulfamethoxazole	20 – 320 µg/mL

Table S3. Antimicrobial susceptibility profiles Gram-positive cocci subjected to AST.

Lab no.	Organism	Cefoxitin Screen	Oxacillin	Cefuroxime	Cefuroxime Axetil	Gentamicin High Level (synergy)	Gentamicine	Ciprofloxacin	Moxifloxacin	Inducible Clindamycin Resistance	Erythromycin	Telithromycin	Clindamycin	Linezolid	Daptomycin	Teicoplanin	Vancomycin	Tetracycline	Nitrofrantoin	Fusidic Acid	Mupirocin	Rifampicin	Trimethoprim/ Sulfamethoxazole	No of categories NS	NS to 0 categories	NS to 1 - 2 categories	MDR (NS to 3 or more)	XDR (NS to 13 or more)	PDR (NS to all 15 categories)
JR1h	*Enterococcus faecalis*					R		R			R			S		R	R	S	R					6	0	0	1	0	0
JR2g	*Enterococcus faecalis*					R		R			R			S		R	R	S	R					6	0	0	1	0	0
JR2h	*Enterococcus faecalis*					R		R			R			S		R	R	R	R					7	0	0	1	0	0
JR2j	*Enterococcus faecalis*					S		I			R			S	S	R	R	S	I					5	0	0	1	0	0
JR3x	*Staphylococcus cohnii cohnii*						S	S	R		R	S	S			I	S	S	S	R		S	R	5	0	0	1	0	0
JR5k	*Staphylococcus haemolyticus*		R				R	R	R		S	S	R			I	I	R	S	S		S	R	8	0	0	1	0	0
JR5l	*Staphylococcus cohnii cohnii*						S	I	R		R	S				I	S	S	I	R		S	R	6	0	0	1	0	0
JR8f	*Staphylococcus warneri*		R				S	R	R		R				S			R					S	4	0	0	1	0	0
																									0	0	8	0	0

S	Susceptible
I	Intermediately resistant
R	Resistant

Table S4. Antimicrobial susceptibility profiles Gram-negative bacilli subjected to AST.

Lab no.	Organism	Ampicillin	Amoxicillin/ Clavulanic acid	Piperacillin/ Tazobactam	Cefuroxime	Cefuroxime/ Axetil	Cefoxitin	Cefotaxime	Ceftazidime	Cefepime	Ertapenem	Imipenem	Meropenem	Amikacin	Gentamicin	Tobramycin	Ciprofloxacin	Tigecycline	Colistin	Trimethoprim/ Sulfamethoxazole	No of categories NS	NS to 0 categories	NS to 1 - 2 categories	MDR (NS to 3 or more)	XDR (NS to 11 or more)	PDR (NS to all 13 categories)
JR1a1	*Aeromonas sobria*		S	S	S		S		S	S		S		S	S		S	S		S	0	1	0	0	0	0
JR1a2	*Aeromonas hydrophila/caviae*		S	S	S		S		S	S		S		S	S		S	S		S	0	1	0	0	0	0
JR1b	*Aeromonas hydrophila/caviae*		I	S	R		S		S	S		S		S	S		S	S		R	3	0	0	1	0	0
JR1c	*Aeromonas sobria*		R		R		R		S	S		S		S	S		S	S		S	3	0	0	1	0	0
JR1g	*Kluyvera ascorbata*	R	R	S	R	R	I	R	R	S	S	I	I	S	S	R	S	S	S	R	8	0	0	1	0	0
JR1i	*Pseudomonas stutzeri*			S				S	S	S		S	S	I	S	S	S	S		R	2	0	1	0	0	0
JR1j1	*Escherichia coli*	R	R	R	R	R	S	R	R	R	S	S	S	I	I	R	R	S	S	R	8	0	0	1	0	0
JR1j2	*Escherichia coli*	R	R	R	R	R	S	R	R	R	S	I	S	I	I	R	R	S	S	R	9	0	0	1	0	0
JR2a	*Pseudomonas aeruginosa*			R				R	R	R		S	S	S	S	S	S	R	S		3	0	0	1	0	0
JR2c	*Aeromonas hydrophila/caviae*		R	S	R		R		R	S		S		S	S		S	S		S	4	0	0	1	0	0
JR2d	*Aeromonas hydrophila/caviae*		I		S		S		S	R		I		S	S		S	S		S	3	0	0	1	0	0
JR2e	*Aeromonas sobria*		S		S		S		S	S		I		S	S		S	S		S	1	0	1	0	0	0
JR2k	*Escherichia coli*	R	R	R	R	R	R	R	R	R	I	I	R	S	S	S	R	S	S	R	9	0	0	1	0	0
JR3a	*Escherichia coli*	R	R	R	R	R	R	R	R	R	I	I	I	S	R	R	R	S	S	R	10	0	0	1	0	0
JR3c	*Escherichia coli*	R	R	R	R	R	R	R	R	R	I	I	I	I	S	R	R	S	S	S	9	0	0	1	0	0
JR3f	*Citrobacter braakii*		R	R	R	R	R	R	R	R	S	R	S	I	R	R	S	S	S	S	7	0	0	1	0	0
JR3g	*Aeromonas sobria*		I		S		S		S	S		I		S	S		S	S		S	2	0	1	0	0	0
JR3i	*Aeromonas hydrophila/caviae*		S	S	S		S		S	S		S		S	S		S	S		S	2	0	1	0	0	0
JR3p	*Raoultella planticola*	R	S	S	R	R	S	S	I	S	S	R	R	S	S	S	S	S	S	S	4	0	0	1	0	0
JR4a	*Escherichia coli*	R	R	R	R	R	R	R	R	R	I	I	R	S	R	R	R	S	S	R	10	0	0	1	0	0
JR4b	*Acinetobacter baumannii complex*			R				R	S	S		S	S		S	S	S	S	S	R	3	0	0	1	0	0
JR4d2	*Citrobacter braakii*		R	R	R	R	R	R	R	R	S	I	S	I	R	R	S	S	S	S	7	0	0	1	0	0
JR4h	*Enterobacter asburiae*		R	I	R	R	R	S	R	S	S	R	S	S	S	S	I	S	S	R	8	0	0	1	0	0
JR5d	*Shewanella putrefaciens*			S				S	S	S		R	R	S	S	S	S	S	R	S	2	0	1	0	0	0
JR5i	*Serratia plymuthica*		S	S	S	S	S	S	R	S	S	S	S	S	S	S	S	S	R	R	3	0	0	1	0	0
																						2	5	18	0	0

S Susceptible
I Intermediately resistant
R Resistant
